# Epidermólisis ampollosa con atresia pilórica: reporte de dos casos en hermanos consecutivos

**DOI:** 10.7705/biomedica.5401

**Published:** 2021-06-15

**Authors:** Katherine Márquez, Diego Andrés Rodríguez, Luis Alfonso Pérez, Mauricio Duarte, Luis Augusto Zárate

**Affiliations:** 1 Servicio de Pediatría, Hospital Universitario de Santander, Bucaramanga, Colombia https://orcid.org/0000-0002-3094-5692 Hospital Universitario de Santander Bucaramanga Colombia; 2 Servicio de Pediatría, Hospital Universitario de Santander, Bucaramanga, Colombia Hospital Universitario de Santander Bucaramanga Colombia; 3 Servicio de Pediatría, Hospital Universitario de Santander, Bucaramanga, Colombia Hospital Universitario de Santander Bucaramanga Colombia; 4 Servicio de Pediatría, Hospital Universitario de Santander, Bucaramanga, Colombia Hospital Universitario de Santander Bucaramanga Colombia; 5 Servicio de Pediatría, Hospital Universitario de Santander, Bucaramanga, Colombia Hospital Universitario de Santander Bucaramanga Colombia

**Keywords:** epidermólisis ampollosa, recién nacido, obstrucción intestinal, Epidermolysis bullosa, infant, newborn, intestinal obstruction

## Abstract

La atresia pilórica es una malformación digestiva poco frecuente y representa alrededor del 1% de las atresias intestinales. En el 55% de los casos, se asocia con alguna otra alteración genética o anatómica, especialmente la epidermólisis ampollosa, que se presenta en el 20% de ellos, en una asociación que se describe como un síndrome de mal pronóstico.

Se presentan dos casos de hermanos consecutivos con esta condición, ambos con un desenlace fatal. Se hizo, además, una revisión de la literatura y se expusieron los puntos más importantes.

## Abstract (en)

Pyloric atresia is a rare digestive malformation. It represents about 1% of intestinal atresias and is associated with some other genetic or anatomical alteration in 55% of the cases. In 20% of them, it is associated with epidermolysis bullosa, which is described as an established syndrome with a bad prognosis.

We present two cases of consecutive siblings with this condition and fatal outcomes in both of them. We made a review of the literature and discussed the main topics.

La atresia pilórica es una malformación digestiva poco frecuente descrita por primera vez en 1749. Representa alrededor del 1% de las estenosis intestinales y ocurre en menos de uno por cada 100.000 nacidos vivos (1,2). En 55% de los casos puede asociarse con alteraciones genéticas o anatómicas (3) y, en 20% de ellos, lo hace con la epidermólisis ampollosa (4). Esta asociación se describe como un síndrome establecido, con cerca de 100 casos reportados en la literatura (5). Fue descrita por primera vez en 1948 y era conocida antes como síndrome de Carmi (2,6).

En general, se considera de mal pronóstico por la gravedad del compromiso cutáneo y respiratorio, la sobreinfección y las complicaciones hemodinámicas e hidroelectrolíticas derivadas (7-9).

Se presentan los casos de dos hermanos consecutivos con epidermólisis ampollosa y atresia del píloro al nacer; se hace énfasis en el cuadro clínico y las cirugías llevadas a cabo.

## Presentación de casos

### Caso 1

Se trata de un paciente nacido por cesárea a las 39 semanas de gestación, fruto de un segundo embarazo de padres no consanguíneos, a quien se le hizo diagnóstico de polihidramnios a las 34 semanas.

Fue llevado a consulta a los cuatro días de haber nacido por presentar vómito alimentario desde el nacimiento, que resultó en una pérdida del 13% de su peso. El bebé se había alimentado exclusivamente con leche materna y desde el primer día de vida su madre notó lesiones vesiculares en múltiples regiones de su cuerpo.

Fue hospitalizado por deshidratación hipernatrémica y lesiones vesiculares en la piel del abdomen, manos y codos. Inicialmente, se consideró una sepsis temprana y se inició el tratamiento antibiótico contra *Staphylococcus aureus*. Fue valorado por la Sección de Cirugía Pediátrica y allí se sospechó una atresia del píloro mediante el examen clínico, a raíz de la epidermólisis ampollosa asociada con vómito.

Se solicitó una radiografía de abdomen, la cual evidenció la acentuada distensión de la cámara gástrica sin paso distal del gas. Al día siguiente, fue sometido a una piloroplastia mediante laparotomía para corregir la atresia antropilórica de tipo I. El bebé evolucionó apropiadamente y, al quinto día de la operación, toleró la vía oral con adecuado tránsito intestinal; la nutrición parenteral se suspendió al séptimo día de la intervención.

En la valoración de dermatología, se ratificó el diagnóstico de epidermólisis ampollosa y se recomendó el tratamiento tópico con barreras de protección, gasas tratadas con vaselina y antibiótico.

A los 16 días de vida (día 11 del posoperatorio), el paciente presentó episodios de vómito y en la radiografía de vías digestivas altas practicada se apreció falta de paso del medio de contraste en el sitio de la anastomosis del píloro.

En la segunda laparotomía, se encontraron múltiples bridas y obstrucción de la anastomosis por una válvula pilórica posterior, por lo que se procedió a practicar una nueva anastomosis antroduodenal. A los dos días de esta nueva intervención, presentó deterioro clínico y de los indicadores paraclínicos, por lo cual se inició un tratamiento antibiótico de tercera línea (vancomicina más meropenem).

Tres días después, se reinició de forma progresiva la alimentación por vía oral hasta lograr la tolerancia completa. Finalmente, a los 30 días de vida del paciente, se autorizó el egreso hospitalario y se le dieron a la madre las indicaciones sobre el cuidado de la piel y los signos de alarma por los que debía volver a consulta si se presentaban.

Dos días después, la madre llevó el bebé a consulta por tos, cianosis y dificultad respiratoria acompañada de síntomas de obstrucción bronquial, ante lo cual se hospitalizó con diagnóstico de bronquiolitis aguda y sospecha de infección por *Bordetella* spp. A los siete días de su ingreso, presentó deterioro clínico con evidencia de consolidación neumónica, por lo que se inició el tratamiento antibiótico. Ante la falta de mejoría, se decidió administrarle antibióticos de tercera línea más oseltamivir en la unidad de cuidados intensivos.

En el estudio de la deglución mediante videofluoroscopia, se demostró un trastorno grave con importante broncoaspiración, por lo cual se diagnosticó neumonía por aspiración. Se consideró practicarle una gastrostomía, pero el paciente presentó falla respiratoria asociada con estridor y, por ello, se optó por la nutrición parenteral total.

A los dos meses de vida, presentó nuevamente falla respiratoria por obstrucción de las vías altas asociada con neumonía y, a los tres días de su ingreso a la unidad de cuidados intensivos pediátricos, tuvo falla multiorgánica, choque resistente al tratamiento y falla renal anúrica y, finalmente, falleció.

Durante la hospitalización se le explicó a la madre la naturaleza genética de la enfermedad, lo cual determinaba su mal pronóstico, y se le planteó la importancia de la asesoría genética en caso de planear otro embarazo.

### Caso 2

**Figura 1:**
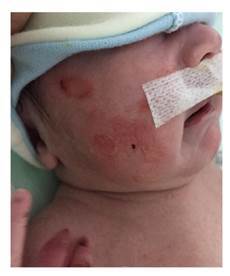
Múltiples lesiones ampollosas en la piel con zonas esfaceladas

Se trata de un paciente fruto del tercer embarazo de padres no consanguíneos -los mismos individuos del primer caso presentado-, nacido por cesárea a las 37 semanas de edad gestacional con un peso de 3.500g. Fue hospitalizado en su primer día de vida por distensión abdominal y vómitos abundantes, así como múltiples ampollas en la piel, sin evidencia de aplasia cutis (figura 1).

En los antecedentes perinatales se había reportado polihidramnios y atresia de píloro según la ecografía, así como un hermano fallecido debido a la asociación de atresia de píloro y epidermólisis ampollosa.

**Figura 2:**
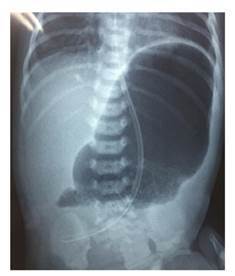
Radiografía de tórax y abdomen. Se observa la gran burbuja de aire en la cámara gástrica.

**Figura 3:**
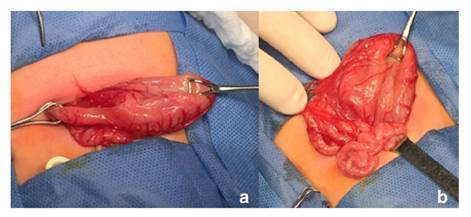
Hallazgos en la cirugía. a. Se observa el cordón fibroso en la región pilórica. b. Asas del intestino delgado de pequeño calibre

En la radiografía de abdomen practicada, se observó distensión por aire de la cámara gástrica, sin paso distal de gas (figura 2). El bebé fue sometido a cirugía en su tercer día de vida; en la intervención se encontró una banda fibrótica en la región del píloro, la cual se extendía desde el antro gástrico hasta la primera porción del duodeno, correspondiente a una atresia pilórica de tipo 2 (figura 3). Se le practicó una anastomosis gastroyeyunal término-lateral (Y de Roux). Posteriormente, el paciente presentó falla respiratoria aguda, por lo que se le ingresó en cuidados intensivos durante seis días; el tránsito intestinal fue lento y se le comenzó a alimentar por vía oral con pequeñas porciones.

A los diez días de vida, el bebé presentó sepsis neonatal tardía por *Enterobacter cloacae* y recibió un antibiótico de amplio espectro. Asimismo, presentó estridor inspiratorio, el cual mejoró con el tratamiento médico, y se le dio egreso a los 25 días.

A los dos meses de vida, el paciente reingresó al hospital con un cuadro clínico de fiebre, desnutrición, hiponatremia, hiperpotasemia e hipoalbuminemia. En la radiografía de tórax vertical, se evidenció neumoperitoneo y, en la ecografía, líquido espeso libre en la cavidad abdominal. Ante la sospecha de una perforación intestinal, se le practicó una laparotomía exploratoria en la cual se encontró perforación en el intestino delgado y en el sigmoides, por lo que requirió resección intestinal y colostomía.

El paciente ingresó en malas condiciones a la unidad de cuidados intensivos pediátricos después de la cirugía y requirió transfusión, administración de inotrópicos y agentes vasopresores, así como asistencia respiratoria. Allí desarrolló una neumonía bilateral y falleció a los tres meses de vida por falla respiratoria aguda.

### Consideraciones éticas

Se contó con el consentimiento informado para la publicación de los casos clínicos y las fotografías.

## Discusión

La atresia pilórica se produce en la quinta semana de gestación por falta de recanalización duodenal; es una condición autosómica recesiva que se puede heredar en uno de cada cuatro hijos (4). Existen tres tipos de atresia pilórica: el tipo 1, o de membrana; el tipo 2, en el que se presenta un cordón fibrótico, y el tipo 3, con desconexión completa del estómago y el duodeno (10).

En la estenosis del píloro se puede presentar polihidramnios, vómito no bilioso desde el nacimiento y distensión muy importante del estómago en la radiografía de abdomen, lo cual la diferencia de la atresia duodenal, en la cual el vómito es usualmente de características biliosas y el estómago muestra el signo de la doble burbuja en la radiografía (11). Generalmente, la corrección quirúrgica del defecto se hace mediante una reconstrucción del tipo Billroth I por laparotomía, aunque hay reportes de corrección laparoscópica del defecto con menor morbilidad y mortalidad (11).

La epidermólisis ampollosa integra un raro grupo de alteraciones genéticas con fenotipos diversos (12,13) y ocurre en dos de cada 1’000.000 de nacidos vivos (2). Se caracteriza por fragilidad cutánea con formación de vesículas en la piel derivadas de pequeños traumas, y se categoriza por microscopía electrónica, según la profundidad de la separación de la membrana basal, en tres grupos: simple, de la unión (dermoepidérmica) y distrófica (7). Algunos describen dos grupos adicionales con hemidesmosomas: uno asociado con distrofia muscular y otro con estenosis del píloro (7). Asimismo, se han identificado mutaciones de las proteínas de la unión dermoepidérmica y la dermis papilar superior (7).

La atresia pilórica y la epidermólisis ampollosa son trastornos autosómicos recesivos poco comunes, y su asociación, reconocida por primera vez en 1964 por Rilke, *et al.* (2), representa un reto diagnóstico y terapéutico. Se piensa que el desarrollo de la estenosis del píloro en pacientes con epidermólisis ampollosa es secundaria a la separación de la mucosa con fibrosis progresiva del canal pilórico (2,5). Su origen fisiopatológico no está claramente descrito, pero se menciona que los defectos en la expresión de las integrinas, que causan las lesiones dérmicas típicas de la epidermólisis, pueden relacionarse también con la formación de cicatrices en el canal pilórico, las cuales pueden ocasionar un proceso de estenosis (7).

Su diagnóstico prenatal es posible con ultrasonido, con el cual pueden encontrarse anormalidades asociadas con la epidermólisis ampollosa y hallazgos indicativos de atresia pilórica, como dilatación de la cámara gástrica, presencia de partículas sólidas en el líquido amniótico conocidas como signo de “copos de nieve”, que corresponderían a la descamación epidérmica (4,14), además de un novedoso signo que es la separación completa de la membrana corioamniótica (8). También, es posible el estudio genético de la biopsia de las vellosidades coriónicas, pero este implica una mayor morbilidad (15). Hay, asimismo, un reporte anecdótico de diagnóstico prenatal por resonancia magnética fetal (4) y se ha descrito la elevación de la alfafetoproteína materna (2).

Cuando el diagnóstico es prenatal, está indicado el nacimiento por cesárea para disminuir el trauma cutáneo al paso por el canal de parto. Se manifiesta clínicamente desde el nacimiento o en los primeros días de vida, con lesiones cutáneas y vómitos alimentarios, y el diagnóstico puede sospecharse si se encuentra la asociación, aunque a veces puede ser necesaria la biopsia cutánea (15).

En los dos pacientes que se presentan, las manifestaciones de la enfermedad fueron tempranas; sin embargo, el diagnóstico prenatal del segundo caso no se documentó adecuadamente debido, en gran medida, al desconocimiento de la enfermedad por parte de la madre, a pesar de las explicaciones dadas por el personal de salud en el primer caso.

El pronóstico de la atresia pilórica es bueno cuando se hace una corrección quirúrgica adecuada, pero, es malo cuando se trata de una atresia pilórica con epidermólisis ampollosa, al punto que algunos cuestionan la pertinencia de la corrección quirúrgica (4). Esta última se considera una condición mortal, aunque se han reportado casos de supervivencia hasta los 14 y 16 años (5). La tendencia actual parece ser operar a los neonatos estables y descartar la cirugía en los inestables o en aquellos con alguna comorbilidad adicional (1). La corrección quirúrgica depende del tipo de atresia pilórica: en la de tipo 1, se interviene la membrana y se hace una piloroplastia; en la de tipo 2, se reseca el segmento atrésico y se hace una gastroduodenostomía, y en la de tipo 3, se usa la gastroduodenostomía (5).

La evolución inmediata de la intervención quirúrgica en los dos casos fue aceptable; sin embargo, el segundo paciente tuvo que ser intervenido nuevamente estando en malas condiciones. Llama la atención el hallazgo de una perforación intestinal baja, lo que no ha sido descrito en esta condición, a diferencia de las perforaciones en el área de estenosis (16).

La relación entre la epidermólisis ampollosa con atresia pilórica y la genética del individuo se reconoció por primera vez en la década de 1970, y condujo a la exploración y el estudio de las características genéticas responsables, lográndose identificar en ese momento los genes *ITGA6*, *ITGB4* y PLEC (12). El asesoramiento genético es crucial para las familias con una historia de epidermólisis ampollosa con atresia pilórica. Las pruebas genéticas moleculares de los padres permiten establecer el estado de portador heterocigoto. Además, como la epidermólisis ampollosa con atresia pilórica es un trastorno autosómico recesivo, existe la probabilidad de que la enfermedad se presente en uno de cada cuatro embarazos cuando ambos padres son portadores obligados.

En este reporte, la asociación de epidermólisis ampollosa con atresia pilórica se presentó en dos hermanos consecutivos, pero, aunque se le indicó a la madre la valoración genética desde el primer caso, ella no continuó con el seguimiento, lo que impidió hacer el diagnóstico genético y asesorarla sobre el pronóstico de un nuevo embarazo.

En conclusión, la atresia pilórica debe considerarse siempre en los casos de examinar un neonato con vómito no bilioso y, además, debe contemplarse la epidermólisis ampollosa, incluso, cuando no haya alteraciones cutáneas. El tratamiento y el pronóstico de la presentación simultánea de estas dos condiciones deben evaluarse de manera multidisciplinaria y discutirse con los padres.
